# Supplementary material for: Content-rich biological network constructed by mining PubMed abstracts
Source: BMC Bioinformatics. 2004 Oct 8;5:147. doi: 10.1186/1471-2105-5-147 (PMC528731; doi:10.1186/1471-2105-5-147)
Supplement: Additional File 2 — The original results of the above study (non-essential files are deleted to keep the file size under the limit set by BMC bioinformatics). [file 1471-2105-5-147-S2.bz2 › chilibotAdditionalFile2/dip05/698ID9287012E18025/html/FTSZ_FTSA.html]

 


 **FTSZ** and **FTSA** 
  
Found 107 abstracts in PubMed, retrieved 05.  
 

 What does Google say? 
 PDF only 
| .edu only 

---

**Interactive relationship** (e.g. stimulation, inhibition, etc)

**Neutral relationship**- These results suggest that the molecular mechanism of the  **FtsA** \* bypass is to stabilize  **FtsZ**  assembly via a parallel pathway.  Ref: 12634424 Proc Natl Acad Sci U S A, 2003
- **FtsA**  and ZipA localized into a division ring following the same pattern and time course as  **FtsZ** .  Ref: 12754232 J Bacteriol, 2003

**Non-interactive relationship** (e.g. studied together, co-existance, homology, etc.)

- Concentration and Assembly of the Division Ring Proteins  **FtsZ** ,  **FtsA** , and ZipA during the Escherichia coli Cell Cycle.  Ref: 12754232 J Bacteriol, 2003
- By the coexpression of the E. coli  **ftsA**  and  **ftsZ**  genes, which encode key proteins in cell division, growth of recombinant strains as well as production of human leptin and human insulin like growth factor I was improved.  Ref: 12571061 Appl Environ Microbiol, 2003
- Observation of cell morphology revealed that the coexpression of the  **ftsA**  and  **ftsZ**  genes successfully suppressed filamentation caused by the accumulation of recombinant proteins.  Ref: 12571061 Appl Environ Microbiol, 2003
- ZipA and  **FtsA**  are recruited independently to the  **FtsZ**  cytokinetic ring Z ring and are essential for cell division of Escherichia coli.  Ref: 12634424 Proc Natl Acad Sci U S A, 2003
- The concentration of the cell division proteins  **FtsZ** ,  **FtsA** , and ZipA and their assembly into a division ring during the Escherichia coli B r K cell cycle have been measured in synchronous cultures obtained by the membrane elution technique.  Ref: 12754232 J Bacteriol, 2003
- Specifically, cephalexin acylated PBP3 about 50% faster in a population of dividing cells than in a population of filamentous cells in which division was inhibited by inactivation or depletion of  **FtsZ** ,  **FtsA** , FtsQ, FtsW, or FtsN.  Ref: 12813065 J Bacteriol, 2003
